# Loss of a conserved salt bridge in bacterial glycosyl hydrolase BgIM-G1 improves substrate binding in temperate environments

**DOI:** 10.1038/s42003-018-0167-7

**Published:** 2018-10-17

**Authors:** Dipali Mhaindarkar, Raphael Gasper, Natalie Lupilov, Eckhard Hofmann, Lars I. Leichert

**Affiliations:** 1 Ruhr University Bochum, Fakultät für Medizin, Institute for Biochemistry and Pathobiochemistry, Microbial Biochemistry, Universitätsstr. 150, 44780 Bochum, Germany; 2 Ruhr University Bochum, Faculty of Biology and Biotechnology, Department of Biophysics, Protein Crystallography, Universitätsstr. 150, 44780 Bochum, Germany

## Abstract

Salt bridges are the strongest electrostatic interactions in proteins. They substantially contribute to a protein’s structural stability. Thus, mutations of salt bridges are typically selected against. Here, we report on the evolutionary loss of a highly conserved salt bridge in the GH1 family glycosyl hydrolase BglM-G1. BglM-G1’s gene was found in the bacterial metagenome of a temperate, seasonally cold marine habitat. In BglM-G1, arginine 75 is replaced by a histidine. While fully retaining β-glucosidase activity, BglM-G1 is less heat stable than an H75R variant, in which the salt bridge was artificially re-introduced. However, the *K*_*m*_ toward its substrates was lower in wild type, leading to an overall higher catalytic efficiency. Our results indicate that this loss of the salt bridge leads to higher flexibility in BglM-G1’s active site, trading structural stability at high temperatures, a trait not needed in a temperate, seasonally cold habitat, for a more effective catalytic activity.

## Abstract (web summary)

Dipali Mhaindarkar et al. examine the functional effects of a salt bridge loss in the active site of glycosyl hydrolase Bg1M-G1, found in the metagenome of a seasonally cold marine habitat. They show that the catalytic efficiency is overall higher with the lost salt bridge, trading off with lower thermal stability.

## Introduction

Structural stability is governed by hydrophilic and hydrophobic interactions between the backbone and side chains of individual amino acids in a protein. Hydrogen bonds and van der Waals interactions are often the largest contributors to overall protein stability, but individually, salt bridges can contribute substantial energy to protein folding and stability^1–3^. However, our knowledge about mutations that disrupt or enhance these interactions, and their effect on the stability and function of proteins is derived primarily from lab studies, in which these salt bridges were introduced or removed on purpose^4,5^. Indeed, very few studies have been conducted that look at the naturally occurring mutations in a protein, the β-lactamase TEM1 being a rare exception^6^. Since the typical thermodynamic stability of a protein fold, ∆*G*_folding_ is in the range of −20 to −65 kJ mol^−1^^7^, the destruction of one particularly stabilizing interaction, such as a salt bridge or a hydrogen bond can lead to the complete unfolding of a protein. Thus, in general, most mutations are destabilizing and therefore are evolutionary selected against^8^.

Here we report, in contrast, on a naturally occurring mutation of an otherwise invariant residue in the β-glucosidase BglM-G1 that balances a trade-off between structural stability and enzyme activity. β-glucosidases are enzymes hydrolyzing β-glycosidic bonds in substrates ranging from glycosides, to glucosylated flavonoids, glucosylceramides, and oligosaccharides, releasing nonreducing terminal glucosyl residues (see Ketudat et al.^9^ for a comprehensive review). They are also one of the key enzymes in the bioconversion of cellulosic material^10,11^. The important conserved amino acids in β-glucosidases have been well-defined through comparison of primary and 3D structures^12^. In particular, 37 highly conserved amino acids have been identified that are crucial for the function of proteins from glycoside hydrolase family 1 (GH1)^13^. We studied the impact of the naturally occurring substitution of one of these amino acids. The otherwise invariant arginine at position 75 changed to a histidine in this protein, while the other 36 amino acids deemed necessary for the function and structural integrity of members of this protein family remained unchanged. This mutation decreased stability at high temperatures but increased catalytic efficiency toward its substrates. The molecular basis for these changes was investigated through X-ray crystallography of BglM-G1 and its H75R variant, which re-introduces the otherwise invariant arginine. Our data indicate that the natural mutation to histidine at position 75 leads to the loss of an extensive electrostatic network, including a salt bridge in the active site region and implies that evolution of BglM-G1 traded a decrease in thermal stability for improved catalytic efficiency.

## Results

### The metagenomic protein BglM-G1 is a bona fide β-glucosidase

**Fig. 1:**
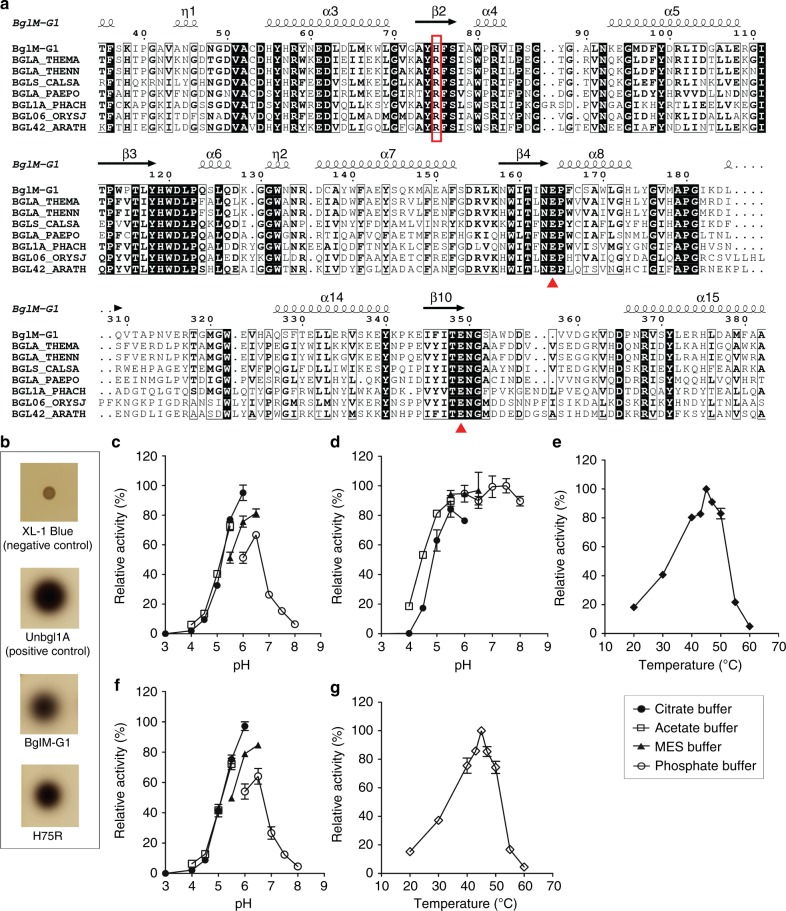
Primary sequence alignment and enzymatic characteristics of BglM-G1 and its H75R variant. **a** Partial alignment of BglM-G1 with homologous GH1 β-glucosidases. BGLA_THEMA: β-glucosidase A from *Thermotoga maritima* (UniProt ID Q08638); BGLA_THENN: 1,4-β-D-glucan glucohydrolase from *Thermotoga neapolitana* (UniProt ID B9K7M5); BGLS_CALSA: β-glucosidase A from *Caldicellulosiruptor saccharolyticus* (UniProt ID P10482); BGLA_PAEPO: β-glucosidase A from *Paenibacillus polymyxa* (UniProt ID P22073); BGLA1A_PHACH: β-glucosidase 1A from *Phanerochaete chrysosporium* (UniProt ID Q25BW5); BGL06_ORYSJ: β-glucosidase 6 from *Oryza sativa subsp. japonica* (UniProt ID Q8L7J2); BGL42_ARATH: β-glucosidase 42 from *Arabidopsis thaliana* (UniProt ID Q9FIW4). A box in red color indicates the position of conserved Arginine 75. Triangles in red color indicate the positions of the catalytic glutamate residues. **b** Esculin-based plate assay testing for β-glucosidase activity in *E. coli* expressing BglM-G1 and H75R. There was no background activity in the *E. coli* XL-1 Blue host. Positive control (Unbgl1A) was a β-glucosidase isolated from a soil metagenomic library (GenBank accession JX566949)^51^. **c** Relative enzyme activity of BglM-G1 over a range of different pHs. Optimum activity is achieved in citrate buffer at pH 6.0. **d** pH stability profile of BglM-G1. **e** Activity of BglM-G1 at different temperatures. Optimum activity is obtained at a temperature of 45 °C. **f** Relative enzyme activity of H75R over a range of different pHs. The optimum pH is virtually identical to wild type. **g** Additionally, the optimum temperature for enzyme activity of H75R was identical at 45 °C. Error bars represent standard deviation, where shown

β-glucosidases of the GH1 are one of the key enzymes in the conversion of cellulose into glucose (for a comprehensive resource for the classification of carbohydrate-active enzymes see the CAZy database^14^). As such, these biocatalysts are essential metabolic enzymes of cellulolytic organisms, as well as tools for the biotechnological conversion of cellulolytic biomass into biofuels and fine chemicals. To identify new β-glucosidases with favorable traits, we searched one of the largest metagenomic datasets, the global ocean sampling (GOS) dataset^15^, for GH1 family members with naturally occurring mutations in otherwise invariant residues. Among the 132 GH1 family members in this dataset, we found only two sequences which featured a substitution of the invariant arginine 75 to a histidine. Both sequences were found in DNA isolates from Chesapeake Bay, Maryland, USA, obtained from very cold water (1 °C) and had a 99% amino acid identity. One of the sequences was otherwise unchanged at the other 36 residues, which are considered highly conserved or invariant in this enzyme family^13^. We selected this protein for further studies and termed it BglM-G1 (GH1 family β-glucosidase of metagenomic origin). Bioinformatic analysis revealed that BglM-G1 exhibited 99% amino acid sequence identity with a β-glucosidase from *Actinobacteria bacterium* BACL2 MAG-120820-bin50 (GenBank accession KRO51423). This sequence was derived by genome assembly from DNA samples obtained from Baltic sea water surface and contains the same mutation of residue 75 to histidine. The closest well-characterized homolog of BglM-G1 was a β-glucosidase from the thermophilic, anaerobic eubacterium *Thermotoga neapolitana* with 50.1% amino acid identity (UniProt accession number P0C946, BGLA_TENN in Fig. 1a)^16^. A conservation analysis of the BglM-G1 sequence using ConSurf^17^ confirmed that arginine at position 75 is indeed invariant in all other known homologs of BglM-G1 (Fig. 1a, Supplementary Fig. 1). To test if BglM-G1 is, despite the mutation of an invariant amino acid, still a bona fide β-glucosidase, we created a synthetic gene *bglM-G1*, which we then could express from a plasmid in *Escherichia coli*. We tested this expression clone for β-glucosidase activity in an esculin-based plate assay. The presence of a black halo surrounding the clone confirmed its β-glucosidase activity (Fig. 1b).

### Enzymatic characteristics of BglM-G1

To biochemically characterize BglM-G1, we expressed a recombinant, N-terminally His_6_-tagged and TEV-cleavage site containing version in *E. coli* and purified it to homogeneity by Ni-NTA affinity chromatography. Activity of this protein was then tested using the chromogenic substrate *p*-nitrophenyl-β-d-glucopyranoside (pNPGlu). BglM-G1 showed optimal activity at pH 6.0 in citrate buffer and had more than 60 % activity in a pH range from 5.0 to 6.5 (Fig. 1c). BglM-G1 was stable in a range from pH 6.0 to 9.0 (Fig. 1d). Its temperature optimum was 45 °C (Fig. 1e), making it by most definitions a mesophilic enzyme. The specific activity of BglM-G1 at optimal pH and temperature was 56.3 ± 1.7 μmol min^−^^1^ mg^−1^ (Table 1), comparable to the activity of other GH1 β-glucosidases^18,19^. Other substrates, toward which BglM-G1 showed high activity were *p*-nitrophenyl-β-d-fucopyranoside (pNPFuc) and *p*-nitrophenyl-β-d-galactopyranoside. Further substrates containing β-glycosidic bonds such as *p*-nitrophenyl-β-d-cellobioside and *p*-nitrophenyl-β-d-xylopyranoside were also hydrolyzed, although the activity towards these substrates was only 4 and 14% of the activity towards BglM-G1’s most preferred substrate, respectively (Table 1). No activity against any of the tested substrates that contained α-glycosidic bonds was detected.

**Table 1:** Specific activity of BglM-G1 and H75R towards aryl-glycosides

Substrate	Specific activity (U/mg)	
	BglM-G1	H75R
*p*-nitrophenyl-β-d-glucopyranoside	56.3 ± 1.7	74.7 ± 1.3
*p*-nitrophenyl-β-d-fucopyranoside	36.9 ± 1.5	80.4 ± 5.3
*p*-nitrophenyl-β-d-galactopyranoside	21.5 ± 0.3	24.1 ± 3.7
*o*-nitrophenyl-β-d-glucopyranoside	17.5 ± 0.7	21.0 ± 0.6
*o*-nitrophenyl-β-d-galactopyranoside	8.8 ± 0.4	9.5 ± 0.5
*p*-nitrophenyl-β-d-cellobioside	7.5 ± 0.1	9.2 ± 0.4
*p*-nitrophenyl-β-d-xylopyranoside	4.8 ± 0.1	6.6 ± 0.5
*p*-nitrophenyl-β-d-mannopyranoside	−	0.2 ± 0.1
*p*-nitrophenyl-α-d-arabinofuranoside	−	−
*p*-nitrophenyl-α-d-rhamnopyranoside	−	−

The specific activities were determined at 45 °C in sodium citrate buffer at pH 6.0

### Structure of BglM-G1 lacks electrostatic interaction network

**Fig. 2:**
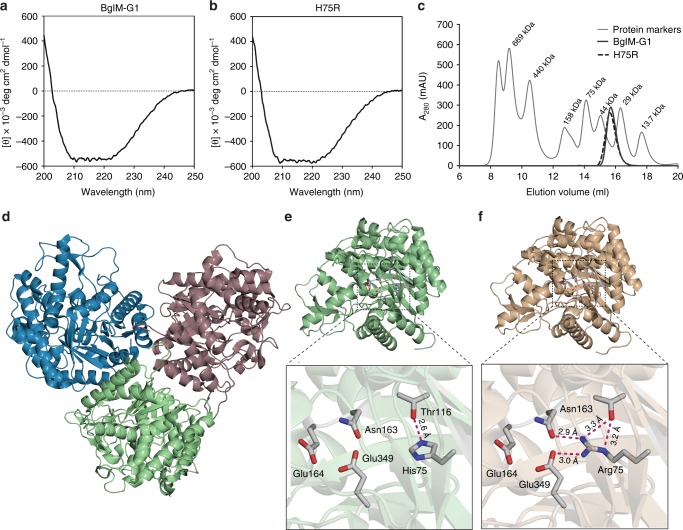
CD spectroscopic analysis and X-ray crystal structure of BglM-G1 and its H75R variant. Far-UV CD spectra of BglM-G1 (**a**) and H75R (**b**) at 20 °C demonstrated overall similarity of the protein structures. Size-exclusion chromatography indicates a monomeric nature of BglM-G1 and H75R (**c**). However, the asymmetric unit of BglM-G1’s crystal structure shows a trimeric assembly (**d**). Examination of the active site of BglM-G1 (**e**) and H75R (**f**) reveals a modified electrostatic interaction network around His75 and Arg75. Electrostatic interactions emanating from residue 75 below 3.5 Å were considered

The fact that BglM-G1 evolved to be a fully active β-glucosidase with a naturally occurring substitution of the otherwise invariant arginine at position 75 provided us with the unique opportunity to study the structural implications of this amino acid in β-glucosidases. We, therefore, re-introduced arginine, replacing the histidine 75 in this enzyme, creating the BglM-G1 variant H75R. This variant showed enzymatic activity (Fig. 1b) and characteristics highly similar to the wild type enzyme with the same pH optimum and buffer preferences (Fig. 1f), optimal temperature (Fig. 1g), and comparable substrate specificities (Table 1). Circular dichroism (CD) spectroscopy demonstrated an overall structural similarity of both wild type (Fig. 2a) and the H75R variant of BglM-G1 (Fig. 2b). For a more detailed structural analysis, we then crystallized both wild type (PDB ID 5NS6) and the H75R variant (PDB ID 5NS7) of BglM-G1 and solved their X-ray structure using data to 1.50 and 1.54 Å, respectively (Table 2). Both BglM-G1 and BglM-G1’s variant H75R crystallized in space group P43 with three monomers in the asymmetric unit (Fig. 2d). Based on size-exclusion chromatography, which showed that both proteins exist in a monomeric form in solution (Fig. 2c), we assume that the trimeric assembly in the crystal is packing-generated. The structure of BglM-G1 was solved by molecular replacement using family 1 β-glucosidase from *Thermotoga maritima*, *Tm*GH1 (PDB ID 1OD0, BGLA_THEMA in Fig. 1a) as a search model. Comparison of the structures of BglM-G1 and *Tm*GH1 monomers revealed a root mean square deviation (RMSD) value of 0.53 Å over 313 Cα atoms and the conformation of the active site glutamates were highly similar (Fig. 2e, f). Upon solving the structure, we observed both in wild type and H75R an unresolved electron density within the active site tunnel. This density does not seem to originate from a peptide chain and we were not able to fit an atomic structure to it. On a sequence level, BglM-G1 and *Tm*GH1 show 49% amino acid sequence identity. Both BglM-G1 and its H75R variant showed a (*β*/*α*)_8_ TIM barrel topology, typical for the GH-A clan including GH1 members^20^. Overall, the side chain and backbone topologies of the amino acid residues in BglM-G1 and its H75R variant were highly similar with an RMSD value of 0.069 Å over 1217 Cα atoms, confirming that the mutation in the active site did not lead to a substantial change in the overall fold. There was, however, a major difference in electrostatic interactions between the mutation site and the active site nucleophile. The naturally occurring histidine at position 75 in BglM-G1 formed only a single hydrogen bond with the backbone oxygen of threonine 116 (Fig. 2e), while arginine at position 75 formed, in addition to hydrogen bond interactions with residues Gln163 and Thr116, a salt bridge with the catalytic nucleophile glutamate at position 349 (Fig. 2f)

**Table 2:** Data collection and refinement statistics for structural datasets

	BglM-G1 (PDB ID 5NS6)	H75R (PDB ID 5NS7)	H75R co-crystallized with 1-deoxynojirimycin (PDB ID 5NS8)
*Data collection*			
Space group	P43	P43	P43
*Cell dimensions*			
*a*, *b*, *c* (Å)	138.02, 138.02, 97.91	137.92, 137.92, 98.04	137.75, 137.75, 97.80
*α*, *β*, *γ* (°)	90.00, 90.00, 90.00	90.00, 90.00, 90.00	90.00, 90.00, 90.00
Resolution (Å)	48.80–1.50 (1.54–1.50)^a^	48.76–1.54 (1.58–1.54)	48.70–1.55 (1.59–1.55)
CC1/2	99.9 (29.9)	99.8 (31.5)	99.9 (39.4)
*R*_sym_ (%)	14.0 (238.2)	10.4 (182.1)	15.4 (233.4)
*I* / σ*I*	13.31 (1.05)	11.28 (0.89)	11.97 (0.80)
Completeness (%)	100.0 (100.0)	99.9 (99.7)	100.0 (100.0)
Redundancy	13.6 (13.7)	6.8 (6.2)	13.7 (13.0)
Wavelength (Å)	1.00	1.00	1.03
*Refinement*			
Resolution (Å)	48.80–1.50 (1.54–1.50)^a^	48.76–1.54 (1.58–1.54)	48.70–1.55 (1.59–1.55)
No. of reflections used	292,649	270,326	263,952
*R*_work_/*R*_free_ (%)	14.8/17.2	15.5/17.2	16.1/17.2
*No. of atoms*			
Protein (non-H)	11845	11625	11612
Ligand/ion (non-H)	193	142	140
Water	859	756	747
*B*-factors			
Protein	22.7	26.25	24.14
Ligand/ion	56.16	62.41	43.66
Water	31.43	32.26	29.14
*Root mean square deviations*			
Bond lengths (Å)	0.006	0.009	0.005
Bond angles (°)	0.833	0.955	0.782

^a^Values in parentheses represent the highest resolution shell

### BglM-G1 is less thermostable due to loss of salt bridge

**Fig. 3:**
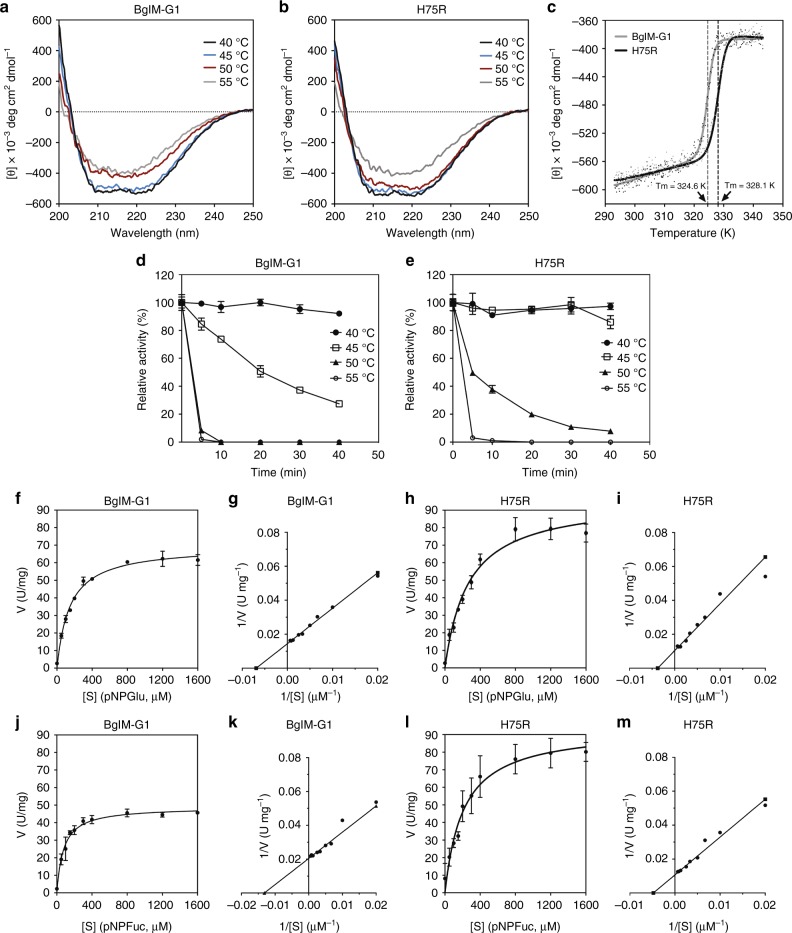
Temperature stability and kinetic parameters of BglM-G1 and its H75R variant. Analysis of the secondary structure of BglM-G1 (**a**) and H75R (**b**) by CD spectroscopy at various temperatures. For BglM-G1 an upward shift in the spectrum, indicative of denaturation of protein, was observed at 50 °C while for H75R a similar spectral transition was observed at 55 °C. Melting curves of the proteins (**c**). The melting temperature (*T*_*m*_) of H75R was almost 3.5 K higher than that of BglM-G1. Thermal stability of BglM-G1 (**d**) and H75R (**e**). Determination of kinetic parameters *K*_*m*_ and *V*_*max*_ of BglM-G1 and H75R at the optimum temperature of 45 °C toward their most preferred substrates and corresponding Lineweaver–Burk plots, pNPGlu (**f**–**i**) and pNPFuc (**j**–**m**). Data based on at least three technical replicates except (**a**–**c**) where duplicates were performed. Error bars represent standard deviation, where shown. **a**, **b** Exemplary figures shown

We hypothesized that this loss of electrostatic interactions should destabilize the structure. Thus, we used far-ultraviolet (UV) CD spectroscopy to investigate temperature-induced structural transitions in both wild-type BglM-G1 and its H75R variant. Both proteins showed highly similar CD spectra with minima between 210 and 220 nm, characteristic for proteins with a significant amount of α-helices^21^. We observed an upward shift in these minima at 50 °C for wild type (Fig. 3a) and 55 °C in the H75R variant (Fig. 3b). The loss in secondary structure in wild type at temperatures lower than in the H75R variant pointed at a lower thermal stability of the former. The similarity in the far-UV CD spectra of the unfolded species also showed a match of the molten-globule state of both wild type and its variant. We then determined the melting temperature of both proteins by monitoring changes in ellipticity at 222 nm, while continuously increasing the temperature from 10 to 80 °C. For BglM-G1 the mid-point of transition was at 51.4 °C (324.6 K with a 95% confidence interval between 324.4 and 324.8 K), while the melting temperature of H75R was at 54.9 °C (328.1 K with a 95% confidence interval between 328.9 and 329.2 K) (Fig. 3c). This difference of almost 3.5 K corresponds to a difference in folding energy of ∆*G* 4.24 kJ mol^−1^ at the melting point. This rather high increase in melting temperature in H75R reflects the structural stability provided by the extensive electrostatic interaction network around residue 75 in β-glucosidases. The differences in melting temperature were also reflected in temperature stability of the enzyme. The variant H75R retained its activity at 45 °C over 40 min, while wild type already lost significant activity (*p* value = 3 × 10^−5^). After 5 min at 50 °C, less than 10% activity was measured in the wild type, while H75R retained almost 50% of its initial activity under these conditions (*p* value = 4 × 10^−6^, Fig. 3d, e).

### Loss of the salt bridge decreases *K*_*m*_ and catalytic efficiency

Because the crystal structure showed that arginine at position 75 forms a strong electrostatic interaction with the active nucleophile glutamate 349, this should lead to a more rigid substrate binding site in the variant. To define the substrate binding site, we co-crystallized the H75R variant with the glucosidase inhibitor 1-deoxynojirimycin^22^, solved the structure with a resolution of 1.55 Å (Table 2), and identified residues that have polar contacts with this sugar derivative. Asparagine 163 and Glutamate 349 were the two residues that had electrostatic interactions with both the inhibitor and Arginine 75 (Supplementary Fig. 2). We thus inspected the B-factor of the atoms in the side chain of these two amino acids. A higher B-factor indicates higher flexibility in the crystal^23^. Indeed, the H75R mutant showed lower normalized B-factor values in these residues, suggesting a more rigid substrate binding site in this mutant (Table 3).

**Table 3:** Average B-factor of the atoms in the side chains of residues Asparagine 163 and Glutamate 349

Protein	Average normalized B-factor Asn163	Average normalized B-factor Glu349	Average normalized B-factor Asn163 and Glu349 combined
BglM-G1	0.93	0.89	0.91
BglM-G1 H75R	0.86	0.85	0.85
BglM-G1 H75R with 1-deoxynorijimycin	0.79	0.82	0.81

The average B-factor of the atoms is normalized to the average B-factor of all atoms in the chain

We tested if such rigidity would lead to changes in the capability to bind substrate. We thus determined the kinetic parameters of both wild type and the H75R variant toward the two most preferred substrates at 45 °C. BglM-G1 had a *k*_*cat*_ value of 55.6 ± 1.5 s^−1^ and a *K*_*m*_ of 135 ± 16 µM toward the pNPGlu substrate. While *k*_*cat*_ of the H75R variant was higher at 99.4 ± 16.3 s^−1^, its *K*_*m*_ was 414 ± 83 µM (Fig. 3f–i, Table 4). These differences are significant with p-values of 0.0042 (*k*_*cat*_) and 0.0014 (*K*_*m*_). This decrease in the substrate affinity lead to an overall lower catalytic efficiency *k*_*cat*_/*K*_*m*_ for the variant of 242 ± 19 s^−1^ mM^−1^ versus 417 ± 44 s^−1^ mM^−1^ for BglM-G1 (*p* value = 0.0002). A similar trend in the differences in the kinetic parameters between the enzymes was observed when pNPFuc was used as substrate (Fig. 3j–m, Table 4) (*p* values are of 4 × 10^−5^ (*k*_*cat*_) and 0.0014 (*K*_*m*_) and 0.0261 (catalytic efficiency). At 10 °C, the differences in *k*_*cat*_ /*K*_*m*_ became less pronounced, but were still significant in the case of pNPFuc as substrate (*p* value = 0.007) (Table 4). At 45 °C, the optimal temperature for the wild-type enzyme, the substitution of Arginine at position 75 in BglM-G1, while decreasing thermal stability, thus increased catalytic efficiency more than 70% toward pNPGlu and more than 50% toward pNPFuc, by enhancing substrate binding. Given the fact that BglM-G1 was isolated from a temperate, seasonally cold environment, the trade-off of lower thermal stability versus higher catalytic efficiency could probably prove overall beneficial.

**Table 4:** Kinetic parameters of BglM-G1 and H75R

Substrate	Enzyme	Temperature (°C)	*k*_*cat*_ (s^−1^)	*K*_*m*_ (µM)	*k*_*cat*_/*K*_*m*_ (s^−1^ mM^−1^)
pNPGlu	BglM-G1	45	55.6 ± 1.5	135 ± 16	417 ± 44
		10	14.9 ± 0.2	72 ± 13	211 ± 38
	H75R	45	99.4 ± 16.3	414 ± 83	242 ± 19
		10	14.2 ± 0.4	71 ± 3	199 ± 4
pNPFuc	BglM-G1	45	41.4 ± 3.0	88 ± 38	519 ± 146
		10	14.4 ± 0.5	90 ± 11	160 ± 16
	H75R	45	72.9 ± 8.1	222 ± 51	335 ± 38
		10	17.5 ± 0.6	154 ± 4	113 ± 2

Kinetic parameters were determined using *p*-nitrophenyl β-d-glucopyranoside (pNPGlu) and *p*-nitrophenyl β-d-fucopyranoside (pNPFuc) as substrates at 45 and 10 °C. Averages and standard deviations are based on at least three technical replicates

## Discussion

In this work, we examined the effects of a naturally occurring loss of a highly conserved network of electrostatic interactions close to the active site of the metagenomic β-glucosidase BglM-G1. This network is emanating from an Arginine, which was previously considered invariant. We identified the lack of the network by obtaining high-resolution X-ray structural data of wild type BglM-G1, in which this Arginine in question has been replaced through evolution with a Histidine. We then compared it to an H75R variant of BglM-G1, in which we re-introduced the Arginine. This revealed an extensive hydrogen bond network including a salt bridge between Arginine 75 and the active site Glutamate 349.

This hydrogen bond network, which is missing in BglM-G1 can be also identified in other structures of β-glucosidases. In fact, a comparison of the published structures of all homologs with at least 30% primary sequence identity to BglM-G1 (39 in total) revealed the salt bridge between Arginine 75 and Glutamate 349 to be present in almost all of these proteins (Supplementary Data 1), except in Strictosidine-O-β-d-glucosidase from *Rauvolfia serpentina* (Serpentine wood) and the psychrophilic β-glucosidase BglU from *Micrococcus antarcticus*, a cold-adapted protein.

The formation of a salt bridge could potentially lead to changes in the ionization of the catalytic nucleophile and thus influence the overall pH optimum of the enzyme. However, a mutational study using the β-glucosidase from *Spodoptera frugiperda*, substituting the Arginine corresponding to Arginine 75 with a Methionine was shown to increase the pH optimum of the enzyme only slightly from 6.2 to 6.5^24^, in line with our observations.

Nevertheless, as expected, this loss of electrostatic interactions led to a substantial loss of structural stability. This manifested itself in a decreased stability and melting temperature of the wild type when compared to the H75R variant. The observed change in melting temperature of 3.5 K is considerably higher than the increase of 1–2 K that is typically achieved by stabilizing mutations in proteins^25^. Although substantially higher increases in the melting temperature have been achieved, these are often the direct effect of hydrophobic interactions or the removal of unfavorable hydrophobic residues on the solvent-exposed surface of proteins^26–28^. While salt bridges certainly stabilize hyperthermophilic proteins^28^, it has been suggested that the stability of mesophilic proteins often does not profit from salt bridges, as the energy required for desolvation is not larger than the energy provided by the electrostatic interactions^29^. Consequently, the replacement of amino acids involved in salt bridges by amino acids that engage in hydrophobic interactions led to substantial stabilization of the Arc repressor^30^. However, other theoretical and experimental work shows that while salt bridges can be both stabilizing and destabilizing, the majority seems to have a stabilizing effect^5,31^, in line with our observations.

Importantly, this loss in thermal stability coincided with an increased catalytic efficiency of BglM-G1, especially at 45 °C, potentially providing a selective advantage. This is in contrast to what would typically be expected from the substitution of a highly conserved amino acid. For example, the aforementioned substitution of the Arginine corresponding to Arginine 75 with a Methionine in the β-glucosidase from *S. frugiperda* resulted in a massive 500-fold decrease in the turnover of the substrate^24^. Depending on the temperature of their environment, enzymes evolve to achieve the optimal balance between the structural rigidity necessary for stability and the flexibility important for substrate binding and enzyme activity^32–34^. The increased ligand binding affinity in the wild-type protein would increase the catalytic efficiency by optimizing substrate binding in an environment where the amount of substrate is scarce. In some cold-adapted enzymes secreted from marine microorganisms, a low *K*_*m*_ has already been implied with the need to acquire more substrate in low substrate concentration environments (see Siddiqui and Cavicchioli^35^ for a comprehensive review). Furthermore, it has been suggested that amino acid changes that destabilize protein structure are involved in the adaptation of enzymes to lower temperatures^36^. The lower B-factors of residues Asparagine 163 and Glutamate 349 in the substrate binding site of BglM-G1’s H75R variant indeed suggest that the higher *K*_*m*_ could be the result of a lower conformational flexibility caused by the stabilizing effect of the electrostatic network formed by Arginine 75 (Table 3). Although atoms in the active sites generally tend to be in low B-factor regions of a protein^37^, it is noteworthy that the average normalized B-factor of these two residues in BglM-G1 is also lower in the majority of its homologs (Supplementary Data 2).

Although the temperature optimum of BglM-G1 classifies it as a mesophilic enzyme, the DNA encoding it was isolated from Chesapeake Bay, Maryland, USA, a seasonally cold marine environment, at only 1 °C^15^. The trade-off in stability versus higher catalytic efficiency is thus probably advantageous to the organism carrying BglM-G1. It should be noted, however, that the gain in catalytic efficiency we observed at 45 °C becomes less pronounced at lower temperatures, closer to the temperatures of the water where the gene encoding BglM-G1 was isolated. However, due to seasonal changes, environmental microbes are typically exposed to greatly varying temperatures. In fact, average maximum surface temperatures in shallow tributaries to Chesapeake Bay can be as high as 30 °C and temperatures above 32 °C have been measured in the vicinity of power plants^38^. Thus, the fitness benefit provided by the substitution of Arginine 75 will be greater at higher temperatures.

Our study implies that while the naturally occurring mutation of the otherwise invariant Arginine 75 in this enzyme class is decreasing thermal stability, it can also lead to higher catalytic efficiency. This evolutionary trade-off is potentially beneficial in the temperate, seasonally cold marine environment where BglM-G1 was discovered.

## Methods

### Bioinformatic sequence analysis

The sequence of hypothetical protein GOS_608286, which we termed BglM-G1, was obtained from the GOS project^15,39^. The J Craig Venter Institute derived the protein sequence (GenBank accession EDH42461.1) from the GOS marine metagenome scaffold JCVI_SCAF_1096626901033 (GenBank accession EP597324.1). The DNA for this scaffold was isolated as part of the GOS project from a sample obtained on December 18, 2003 in Chesapeake Bay, MD, at a depth of 13.2 m and a water temperature of 1 °C^15^.

BlastP analysis of the primary sequence of BglM-G1 was carried out against the NCBI nr and UniProtKB databases. We selected representative sequences with more than 30% amino acid sequence identity to BglM-G1 from UniProtKB from a diverse set of genera for further analysis. A multiple sequence alignment was created using MAFFT (version 7)^40^ and the results visualized using the program ESPript 3.0^41^. Furthermore, the degree of conservation of each amino acid in BglM-G1 was evaluated using the ConSurf server (http://consurf.tau.ac.il/2016/)^17^. To analyze the structural homology of BglM-G1 and the electrostatic interaction network emanating from Arginine 75 in these proteins, BglM-G1 was blasted against the RCSB PDB database. We then selected one structure for any protein with more than 30% amino acid sequence identity, preferably structures with high resolution and without ligand or inhibitor covalently bound to the active site.

### Cloning of BglM-G1 into an expression vector

For the expression of BglM-G1 in *E. coli*, codon optimization of the gene sequence encoding BglM-G1 was carried out using the online tool JCat^42^. The codon-optimized gene sequence was synthesized (GeneArt^®^, Regensburg, Germany) with additional 5′ NdeI and 3′ EcoRI restriction sites. Digested *bglM-G1* was then ligated into pOE, linearized with the same enzymes, for overexpression and purification via His-tag. pOE is a pET-11a (Merck Millipore, Billerica, MA, USA) derivative recently developed in our lab for this purpose^43^. pOE contains an N-terminal 6× His-tag followed by a TEV-protease specific cleavage site. This added the additional N-terminal amino acid sequence MHHHHHHENLYFQGH to BglM-G1 during expression. After TEV cleavage, the scar peptide GH remained. The resultant plasmid pOEbglM-G1 was transformed into *E. coli* BL21 (DE3) for protein expression.

### Site directed mutagenesis

To construct the *H75R* variant of BglM-G1, site directed mutagenesis of pOEbglM-G1 was carried out at position 224 to change the codon CAC to CGC, introducing the corresponding H75R substitution. The oligonucleotides used were: H75R_for: CCAAGCGATAGAGAAGCGGTAAGCACCAACACC and H75R_rev: GGTGTTGGTGCTTACCGCTTCTCTATCGCTTGG. The mutagenic PCR reaction was carried out in a 50 μl reaction mixture containing the following components: 400 μM dNTPs, 1× *Pfu* polymerase buffer, 0.2 μM of each primer (H75R_for and H75R_rev), 100 ng pOEbglM-G1, and 2.5 U *Pfu* polymerase. PCR conditions were: 1 min at 95 °C to denature the template, followed by 12 cycles of 30 s at 94 °C, 30 s at 55 °C, and 7 min at 68 °C. The PCR reaction products were treated with 0.1 U DpnI for 3 h at 37 °C to digest methylated parental DNA, leaving nonmethylated, amplified DNA containing the desired mutation. Competent *E. coli* XL1-Blue cells were transformed with 1 μL of the DpnI-treated PCR product and grown overnight at 37 °C yielding pOEH75R. Successful mutagenesis was confirmed by DNA sequencing (Eurofins Genomics, Ebersberg, Germany).

### Purification of recombinant proteins

For purification of recombinant proteins, *E. coli* BL21(DE3) harboring pOEbglM-G1 and pOEH75R were grown in 1 to 4.5 L of LB media supplemented with ampicillin (100 μg/mL) at 37 °C until cultures reached an optical density OD_600_ of 0.6 ± 0.2. Gene expression was induced at this point by the addition of 1 mM isopropyl-1-thio-β-d-galactopyranoside (IPTG). The culture was then incubated at 20 °C for 16 h. Subsequently, cells were harvested by centrifugation at 4 °C and 7800×*g*. The resulting cell pellet was resuspended in buffer A (50 mM sodium phosphate buffer, pH 8.0 with 300 mM NaCl), one dissolved pill of an EDTA–free protease inhibitor cocktail (Roche, Mannheim, Germany) and 1 mM phenylmethanesulfonyl fluoride. Cells were homogenized by high-pressure cell disruption (Constant systems, Daventry, UK) at 1.9 kbar. Cell debris was removed by centrifugation at 50,000×*g* for 1 h, at 4 °C and the supernatant was filtered through a vacuum filter (Filtropur, Sarstedt, Germany). Protein purification was carried out using an ÄKTApurifier FPLC system (GE Healthcare): the filtrate was loaded on a 5 ml Ni-NTA agarose column (GE Healthcare) pre-equilibrated with buffer A. The column was washed with up to 30-column volumes of buffer A, followed by up to three column volumes with 2% buffer B (50 mM sodium phosphate buffer, pH 8.0 with 300 mM NaCl and 500 mM imidazole) until no further decrease in UV-absorption could be observed. The protein was eluted with a 30-column volume linear gradient running from 2 to 100% buffer B (10 mM to 500 mM imidazole) at a flow rate of 3.0 mL min^−1^. Fractions were analyzed by sodium dodecyl sulfate polyacrylamide gel electrophoresis (SDS–PAGE) and those with highest target protein yield were pooled. To remove the N-terminal 6× His tag, the pooled active fractions were incubated with 6× His-tagged TEV protease (30-fold molar excess of target protein to TEV protease), while dialyzing overnight at 4 °C against buffer A with 1 mM EDTA. The protein solution was transferred to a Falcon tube, MgCl_2_ was added (final concentration 2 mM) and the solution was centrifuged at 6000×*g* for 20 min at 4 °C. To remove the cleaved 6× His-tag and the 6× His-tagged TEV protease, the protein solution was loaded on a Ni-NTA column. SDS–PAGE analysis of the flow-through revealed a single band with a molecular weight of ~50 kDa, which is consistent with BglM-G1’s theoretical mass of 49,874 Da. Fractions of pure protein were collected as flow-through, the buffer exchanged to 50 mM sodium phosphate buffer, pH 7.0 and the protein was concentrated by ultrafiltration using Amicon® Ultra tubes (cutoff 10,000, MWCO, Millipore) and aliquots of concentrated pure protein were stored at −80 °C. Over the course of our experiments we did not observe any loss of activity under these storage conditions. The purity of BglM-G1 and H75R was verified by SDS–PAGE and protein concentration was determined by UV spectroscopy determining the absorbance at 280 nm in 50 mM sodium phosphate buffer (pH 7.0). The extinction coefficient (*ε*_280 nm_) and molecular weight of the purified recombinant protein was calculated based on amino acid composition using the web-based tool ProtParam at ExPaSy (http://web.expasy.org/protparam/)^44^.

### Size-exclusion chromatography

Analytical size exclusion chromatography was carried out with the ÄKTApurifier FPLC-system running UNICORN 5.31 software using a Superdex 200 Increase 10/300 GL column (GE Healthcare). The column was pre-equilibrated with buffer solution (50 mM sodium phosphate, pH 7.0; 150 mM NaCl). The protein solution was loaded onto the column and elution was carried out at a flow rate of 0.4 mL min^−1^. The eluate was analyzed for protein content by online measurement of the absorption at 280 nm. To determine the molecular weight of BglM-G1 and H75R, a mixture of standard calibration protein markers was prepared and 100 μL of the protein mix was loaded on the column. The calibration proteins used were as follows: Thyroglobulin (669.0 kDa), Ferritin (440.0 kDa), Aldolase (158.0 kDa), Conalbumin (75.0 kDa), Ovalbumin (44.0 kDa), Carbonic anhydrase (29.0 kDa), Ribonuclease A (13.7 kDa). The exclusion volume was determined by using Blue Dextran (200,000 kDa).

### Plate-based analysis of β-glucosidase activity

Functional determination of β-glucosidase activity was performed using LB agar plates with 0.1 % esculin hydrate, 0.2 % ferric ammonium citrate, 100 μg mL^−1^ ampicillin and 50 μM IPTG^45^. Clones expressing β-glucosidase activity were identified by observing the plates for appearance of dark halos surrounding the colonies after 24 h of incubation at 37 °C.

### Enzymatic characterization

β-glucosidase activity was determined using pNPGlu as substrate. At least three technical replicates were performed for these experiments. The assay mixture (1 mL) consisting of 2 mM pNPGlu in 50 mM sodium citrate buffer (pH 6.0) was incubated with the enzyme at 45 °C for 10 min. The reaction was stopped by adding 1 mL of 1 M Na_2_CO_3_ and the amount of *p*-nitrophenol released was determined by measuring the absorbance at 400 nm in a JASCO V-650 UV–VIS spectrophotometer running Spectra Manager Version 2 (Jasco, Tokyo, Japan). One unit of enzyme activity was defined as the amount of enzyme releasing 1 μmol of pNP per minute under the above-mentioned assay conditions. Specific activity was expressed in units of active enzyme per milligram of protein.

### Optimum pH, optimum temperature, and temperature stability

The influence of the pH on the activity of BglM-G1 and its H75R variant was determined by measuring the enzyme activity in 100 mM buffers of various pH values (range: 3.0–10.0) with 1 mM pNPGlu as a substrate at 40 °C. At least three technical replicates were performed for each experiment. Buffers used were: 50 mM sodium citrate buffer (pH 3.0–6.0), 50 mM acetate buffer (pH 4.0–5.5), 50 mM MES buffer (pH 5.5–6.5), 50 mM sodium phosphate buffer (pH 6.0–8.0). The pH stability of BglM-G1 was determined by pre-incubation of the enzyme in buffers of varying pHs for 1 h and determining the residual enzyme activity using 100 mM sodium citrate buffer (pH 6.0) at 45 °C. The buffers used were: 50 mM sodium citrate buffer (pHs, 3.0, 4.0, 4.5, 5.0, 5.5, and 6.0), 50 mM acetate buffer (pHs, 4.0, 4.5, 5.0, and 5.5), 50 mM MES buffer (pHs, 5.5, 6.0, and 6.5), and 50 mM sodium phosphate buffer (pHs, 6.0, 6.5, 7.0, 7.5, and 8.0). The enzyme activity without pre-incubation in buffers was considered 100%. The optimum temperature for enzyme activity of BglM-G1 and H75R was determined by carrying out assays at temperatures over a range of 10–60 °C with 2.0 mM pNPGlu in 100 mM sodium citrate buffer (pH 6.0), as this was determined to be the optimal pH buffer. The thermostability of proteins was assessed by incubating enzymes without substrate at various temperatures (10–60 °C) for various time intervals in this buffer and the residual enzyme activity was then determined at 40 °C.

### Substrate specificity

The substrate specificities of BglM-G1 and H75R with various aryl-glycosides were evaluated by incubating 2.0–207.0 pmol of enzymes in a 1 mL reaction mixture with 2 mM substrate in 50 mM sodium citrate, pH 6.0 at 45 °C. Substrate conversion was determined at various time intervals spectrophotometrically by measuring the release of *p*-nitrophenol. In all assays, an appropriate substrate blank was used to account for spontaneous hydrolysis of the substrate. At least three technical replicates were performed.

### CD spectroscopy

Far-UV CD spectra of BglM-G1 and H75R were recorded in a JASCO J-815 (Jasco) coupled to a Peltier temperature control system (PTC 514, Jasco). Protein solutions were measured in quartz cuvettes (Hellma Analytics, Germany) with a path length of 1 mm at concentrations of 2.5–6.0 μM in 10 mM sodium phosphate buffer (pH 7.0). Spectra were continuously recorded between 185 and 250 nm in a 1 mm quartz cuvette at a bandwidth of 2 nm. Thermal unfolding was observed from 10 to 80 °C in 5 K intervals, using temperature interval measurement mode with a gradient of 2 °C/min. Measured ellipticity values, obtained in millidegree (*θ*), were converted to standard units of molar ellipticity [deg cm^2^ dmol^−1^].

### Calculation of the melting point (*T*_*m*_) and folding energy

For melting point determination, data points were obtained continuously at 222 nm from 10 to 80 °C with a ramp rate of 2 °C/min. Two datasets were measured, and the values obtained from both datasets were combined and analyzed by nonlinear regression (Curve fit) in GraphPad Prism (Version 5.0, GraphPad Software, La Jolla, California, USA) using Eq. (1). We assumed a two-state transition of a monomer from folded to unfolded form with an added data correction for pre- and post-transition linear changes in ellipticity as a function of temperature^46^.

1 $$y = a^ \ast \left( {\left( {\varepsilon {\mathrm{F}} + \left( {{\mathrm{CF}}^ \ast T} \right)} \right) - \left( {\varepsilon {\mathrm{U}} + \left( {\mathrm{CU}^ \ast T} \right)} \right)} \right) + \left( {\varepsilon {\mathrm{U}}\left( {\mathrm{CU}^ \ast T} \right)} \right)$$

Here, *ε*F and *ε*U are the mean residual ellipticities of 100% folded and unfolded proteins, respectively, we used the ellipticities −580.7 and −348.0 for BglM-G1 and ellipticities −581.6 and −348.4 for H75R at 25 and 80 °C, respectively, through extrapolation. CF is the linear correction of folded protein as a function of temperature, CU is the linear correction of unfolded protein as a function of temperature and *T* is the absolute temperature (Kelvin). Eq. (1) depends on the fraction of the folded protein α, which is derived by using Eq. (2). The constant of folding (*K*) is dependent on free energy of unfolding (Δ*G*), gas constant (*R*), and temperature (*T*) (Eq. (3)). For each data point a value Δ*G* was derived using Eq. (4). Δ*H* is van’t Hoff enthalpy, ΔCp is heat capacity change upon thermal denaturation, and *T*_*m*_ is the melting temperature.

2 $$a = K/\left( {1 + K} \right)$$

3 $${K} = \exp ^ \ast /\left( { - \Delta G/\left( {R^ \ast T} \right)} \right)$$

4 $$\Delta {G} = \Delta {H}^ \ast (1 - T/T_m) - \Delta {\mathrm{Cp}}^ \ast ((T_m - T) + T^ \ast \ln \;(T/T_m))$$

### Crystallization, data collection, and refinement

The proteins obtained after Ni-NTA affinity chromatography were further purified by size-exclusion using a HiLoad 26/60 Superdex 75 pg column (GE Healthcare), running isocratically with 40 mM sodium phosphate buffer (pH 7.0) and 200 mM NaCl. The eluted fractions from the representative protein peak were dialyzed against 20 mM MES, 50 mM NaCl (pH 7.0) and concentrated (final concentrations: 10.6 mg/mL, BglM-G1 and 8.2 mg/mL, H75R) using Vivaspin 20 10’000 MWCO centrifugal concentrators (Sartorius).

The crystals of BglM-G1 and H75R proteins were obtained by a sitting drop vapor diffusion technique in 2:1 mixture of protein solution:crystallization solution. The crystallization solution used was: 2% (v/v) polyethylene glycol 400, 0.1 M sodium HEPES (pH 7.5), 2.0 M ammonium sulfate. Crystals appeared after 72 h of incubation at 18 °C. The crystals formed were transferred to 1 µL of the reservoir solution with 25% glycerol and flash-cooled in liquid nitrogen. The complex of H75R with 1-deoxynojirimycin was obtained by soaking the inhibitor into pre-existing crystals of H75R. For this, crystals of H75R, obtained after 72 h, were picked and transferred to 10 μL of crystallization solution mixed with 25% glycerol and 6 mM 1-Deoxynojirimycin on a glass cover slide. The cover slide was kept inverted on a well of a 24 well plate containing the crystallization solution. The plates were incubated at 18 °C for 24 h and the crystals were picked and flash-cooled in the liquid nitrogen and stored. X-ray diffraction data of the proteins were collected at the European Synchrotron Radiation Facility (ESRF, Grenoble, France) at beamline ID23-1 at a temperature of 100 K and a wavelength of 1.00 Å (5NS6 and 5NS7) and the DESY PETRA III facility (Deutsches Elektronen-Synchrotron, Hamburg, Germany) at beamline P11 at a temperature of 100 K and a wavelength of 1.03 Å (5NS8). The data were processed using XDS and XSCALE^47^. The structure of BglM-G1 was solved by molecular replacement using PHASER^48^, with coordinates of the closest homolog of BglM-G1, *Tm*GH1 (PDB ID 1OD0)^22^. The structure was refined using phenix.refine^49^ followed by several rounds of manual building using Coot^50^. The structures of H75R and H75R co-crystallized with 1-deoxynojirimycin were solved using the coordinates of the final structure model of BglM-G1. For structures of both proteins, iterative cycles of refinement and manual rebuilding were carried out until the free *R*-factor values converged. Ramachandran statistics were calculated and are as follows (favored/allowed/outliers [%]): BglM-G1 (5NS6) 98.09/1.91/0; H75R (5NS7): 98.17/1.83/0; H75R with 1-deoxynojirimycin (5NS8): 97.79/2.21/0.

The data collection and refinement statistics of the structures are listed in Table 2. The RMSD of structural alignments were determined using the align command in PyMOL. Figures of structures were generated using PyMOL (Schrodinger LLC, New York, NY). Electrostatic interactions were calculated using the distance function of PyMOL with the parameters mode = 2, using a cutoff of 3.5 Å.

### Kinetic analysis

The kinetic parameters of BglM-G1 and H75R were determined using *p*-nitrophenyl-β-d-glucopyranoside (pNPGlu) and *p*-nitrophenyl-β-d-fucopyranoside (pNPFuc) substrates. A substrate concentration in the range of 0.5–5.0 times *K*_*m*_ was used. The reaction velocity of enzymes towards substrates was determined in 100 mM sodium citrate, pH 6.0, at 45 °C and 10 °C. The kinetic constants *K*_*m*_ (mM) and *k*_*cat*_ (1/s) were determined with a nonlinear regression function assuming Michaelis–Menten kinetics for substrate concentration versus velocity in GraphPad Prism (Version 5.0, GraphPad Software).

### Statistical analysis

*p* Values stated were calculated using the *t* test function (two-tailed, two-sample equal variance (homoscedastic)) of Excel for Mac (version 16.16.1 (180814), Microsoft, Redmond, WA). The 95% confidence intervals for the melting points of BglM-G1 and its H75R variant were derived from the fitting data provided by GraphPad Prism.

## Electronic supplementary material

Supplementary Information

Supplementary Data 1

Supplementary Data 2

Description of supplementary data files

## Data Availability

The protein sequence of BglM-G1 (hypothetical protein GOS_608286) can be found under GenBank accession EDH42461.1. Structural data described in this manuscript is deposited in the protein data bank under PDB ID 5NS6, 5NS7, and 5NS8. There are no restrictions on data availability.
