# Supplementary material for: Loss of a conserved salt bridge in bacterial glycosyl hydrolase BgIM-G1 improves substrate binding in temperate environments
Source: Commun Biol. 2018 Oct 17;1:171. doi: 10.1038/s42003-018-0167-7 (PMC6192996; doi:10.1038/s42003-018-0167-7)
Supplement: Supplementary file 1 — Supplementary Information [file 42003_2018_167_MOESM1_ESM.pdf]

Supplementary information

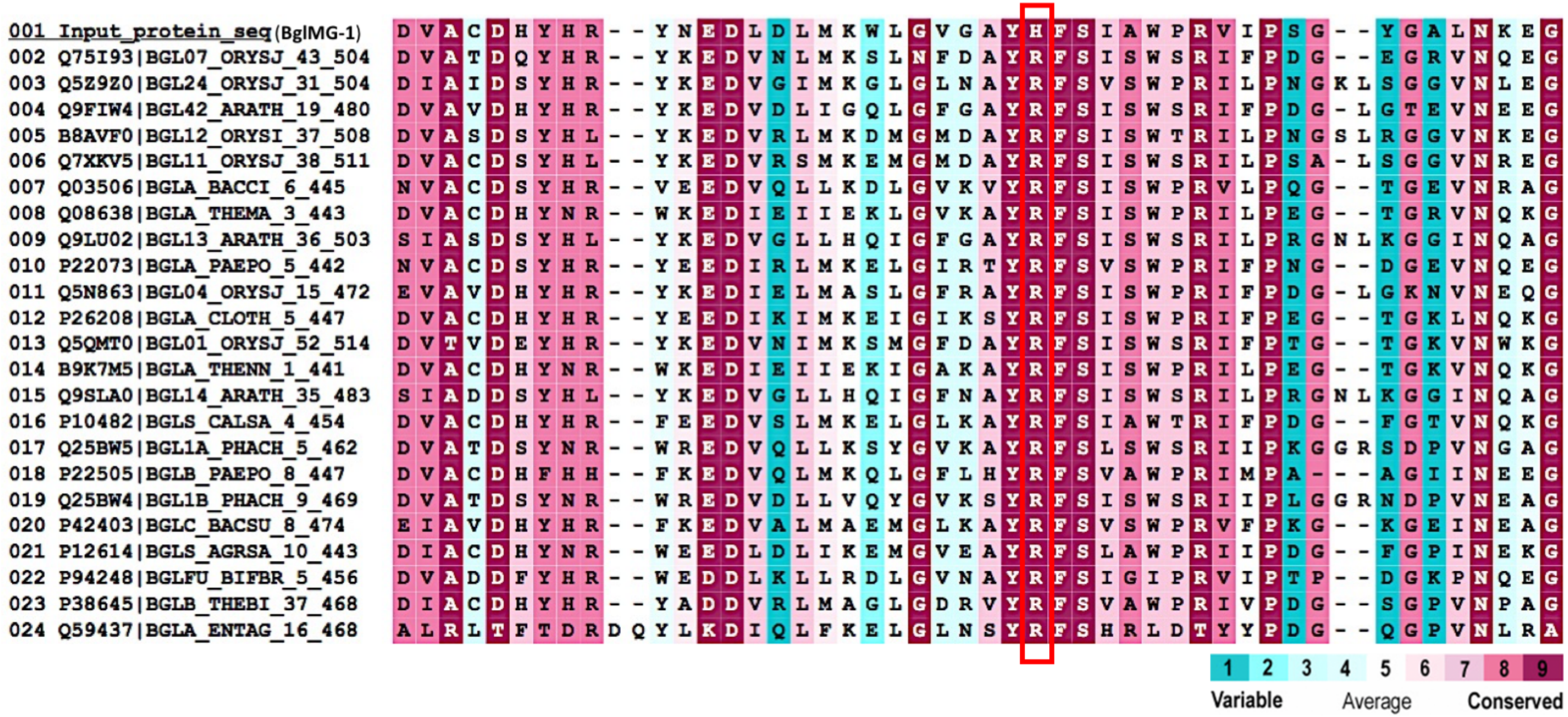

Supplementary Figure 1: Analysis of the evolutionary conservation of amino acids in BglM-G1. A partial sequence alignment of BglM-G1 (Input_protein_seq) carried out using Consurf server[1] with homologous sequences. The conserved arginine at position 75, present in all homologous sequences, is highlighted by a red box. BglM-G1 contains a histidine at this position. The nine-color grade box indicates the conservation score.

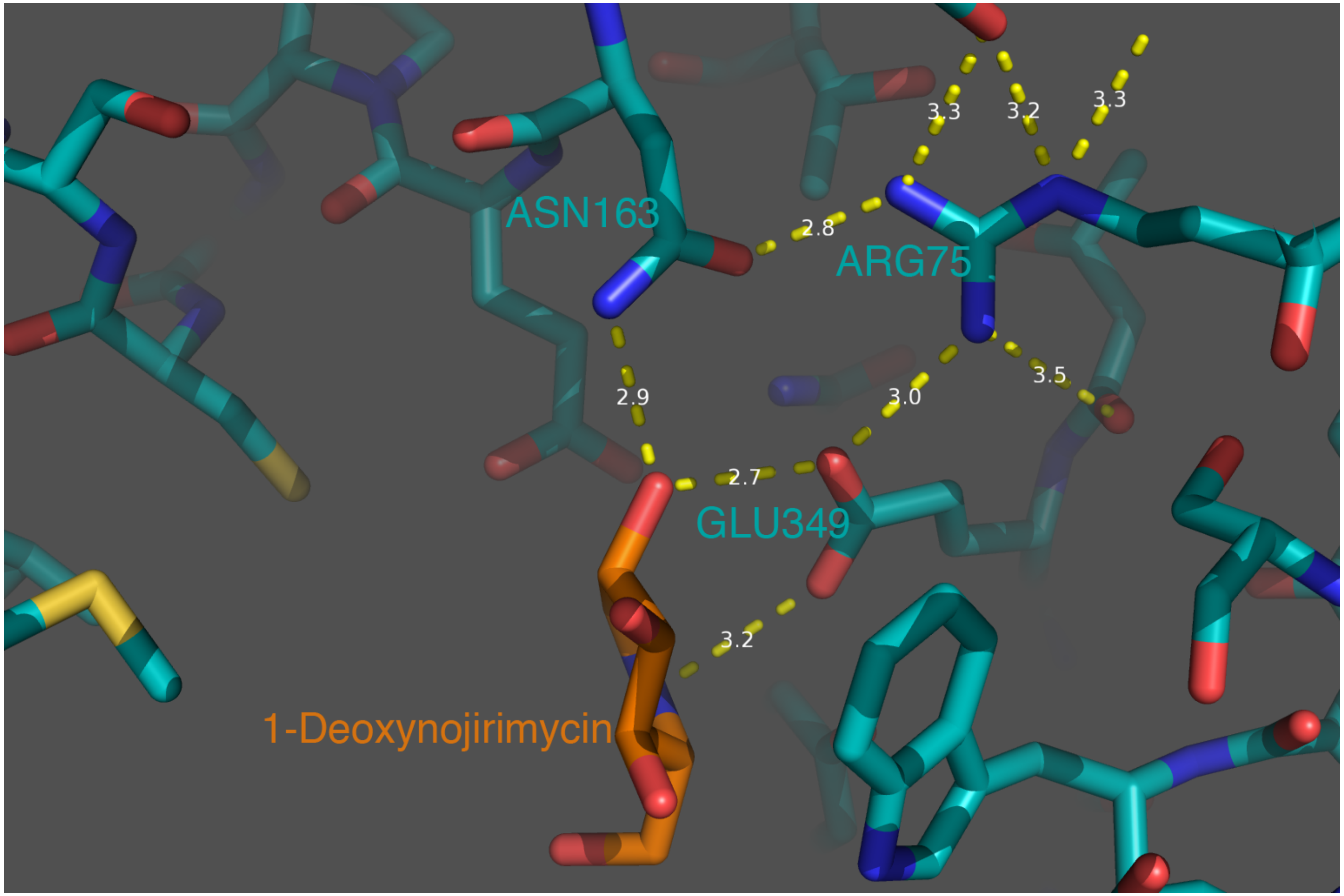

Supplementary Figure 2: Electrostatic interactions between the active site and Arginine 75. Asparagine 163 (Asn163) and Glutamate 349 (Glu349) form electrostatic interactions with both Arginine 75 (Arg75) and the inhibitor 1-Deoxynojirimycin (orange) that binds in the active site of BglM-G1.

Supplementary Data 1: Structural comparison and structural alignments of homologs of BglM-G1. The electrostatic interaction network emanating from residue 75 is highlighted. Alignments were performed using the align command and electrostatic interactions using distance function of PyMOL with the parameter mode=2, setting a cutoff of 4.0 Å.

Supplementary Data 2: B-factors of residues corresponding to Asparagine 163 and Glutamate 349. Average B-factors of atoms of amino acids Asp163 and Glu349 in BglM-G1, its H75R mutant and the corresponding amino acids in its homologs were normalized against the average B-factor of all atoms in the respective chain. Active site with atoms colored by normalized B-factor from low (black) to intermediate (red, orange, yellow) to high (white).

References

1. Ashkenazy, H. *et al.* ConSurf 2016: an improved methodology to estimate and visualize evolutionary conservation in macromolecules. *Nucleic Acids Res.* 44, W344–W350 (2016).
