# Supplementary material for: Loss of a conserved salt bridge in bacterial glycosyl hydrolase BgIM-G1 improves substrate binding in temperate environments
Source: Commun Biol. 2018 Oct 17;1:171. doi: 10.1038/s42003-018-0167-7 (PMC6192996; doi:10.1038/s42003-018-0167-7)
Supplement: Supplementary file 4 — Description of supplementary data files [file 42003_2018_167_MOESM4_ESM.docx]

**Supplementary Data 1: Structural comparison and structural alignments of homologs of BglM-G1.** To analyze the electrostatic interaction network emanating from Arginine 75 in homologs of BglM-G1, BglM-G1 was blasted against the RCSB PDB database. We then selected one structure for any protein with more than 30 % amino acid sequence identity to BglM-G1, preferably structures with high resolution and without ligand or inhibitor covalently bound to the active site. Return values of blastp and structural parameters are listed under “Sequence alignment of BglM-G1 (carried out in RCSB PDB)”. The structures were then aligned to chain A of structure 5NS6 (BglM-G1) using the align command of PyMOL. The structural alignment for each selected homolog is depicted in row “Structural Alignment with BglM-G1 (red: BglM-G1 chain A, teal: Subject)” and the return values of the align command (including RMSD) are listed under “Structure alignment of BglM-G1 (carried out in PyMOL)”. Electrostatic interactions were calculsted using distance function of PyMOL with the parameter mode=2, setting a cutoff of 4.0 Å. The electrostatic interactions are then depicted (row “Electrostatic interactions emanating from residue aligned to H75 in subject”).

**Supplementary Data 2: B-factors of residues corresponding to Asparagine 163 and Glutamate 349.** Average B-factors of atoms of amino acids Asp163 and Glu349 in BglM-G1, its H75R mutant and the corresponding amino acids in its homologs (listed in Supplemental Data 1) were normalized against the average B-factor of all atoms in the respective chain and compared in a table. The active site of BglM-G1 and its homologs is depicted in row “Substrate binding pocket with residues colored by b Factor”, with atoms colored by normalized B-factor from low (black) to intermediate (red, orange, yellow) to high (white).
